# Combined Plasma DHA-Containing Phosphatidylcholine PCaa C38:6 and Tetradecanoyl-Carnitine as an Early Biomarker for Assessing the Mortality Risk among Sarcopenic Patients

**DOI:** 10.3390/nu16050611

**Published:** 2024-02-23

**Authors:** Hung-Yao Ho, Yuan-Ho Chen, Chi-Jen Lo, Hsiang-Yu Tang, Su-Wei Chang, Chun-Ming Fan, Yu-Hsuan Ho, Gigin Lin, Chih-Yung Chiu, Chih-Ming Lin, Mei-Ling Cheng

**Affiliations:** 1 Metabolomics Core Laboratory, Healthy Aging Research Center, Chang Gung University, Taoyuan 333, Taiwan; hoh01@mail.cgu.edu.tw (H.-Y.H.); chijenlo@mail.cgu.edu.tw (C.-J.L.); tangshyu@gmail.com (H.-Y.T.); jimmyf@gap.cgu.edu.tw (C.-M.F.); 2 Clinical Metabolomics Core Laboratory, Chang Gung Memorial Hospital at Linkou, Taoyuan 333, Taiwan; giginlin@cgmh.org.tw (G.L.); pedchestic@gmail.com (C.-Y.C.); 3 Graduate Institute of Biomedical Sciences, College of Medicine, Chang Gung University, Taoyuan 333, Taiwan; david820524@gmail.com; 4 Department of Medical Biotechnology and Laboratory Science, College of Medicine, Chang Gung University, Taoyuan 333, Taiwan; 5 Department of Artificial Intelligence, College of Intelligent Computing, Chang Gung University, Taoyuan 333, Taiwan; shwchang@mail.cgu.edu.tw; 6 Department of Laboratory Medicine, Chang Gung Memorial Hospital at Linkou, Taoyuan 333, Taiwan; 7 Department of Biomedical Sciences, College of Medicine, Chang Gung University, Taoyuan 333, Taiwan; hokylie4@gmail.com; 8 Department of Medical Imaging and Intervention, Chang Gung Memorial Hospital at Linkou and Chang Gung University, Taoyuan 333, Taiwan; 9 Department of Medical Imaging and Radiological Sciences, Chang Gung University, Taoyuan 333, Taiwan; 10 Department of Pediatrics, Chang Gung Memorial Hospital at Linkou and Chang Gung University, Taoyuan 333, Taiwan; 11 Department of Pediatrics, Chang Gung Memorial Hospital at Keelung and Chang Gung University, Taoyuan 333, Taiwan; 12 Division of Internal Medicine, Chang Gung Memorial Hospital at Taipei, Taipei 105, Taiwan; 13 Department of Health Management, Chang Gung Health and Culture Village, Taoyuan 333, Taiwan; 14 College of Medicine, Chang Gung University, Taoyuan 333, Taiwan

**Keywords:** metabolomics, sarcopenia, mortality, DHA, phosphatidylcholine, hypertension

## Abstract

The coming of the hyper-aged society in Taiwan prompts us to investigate the relationship between the metabolic status of sarcopenic patients and their most adverse outcome–death. We studied the association between any plasma metabolites and the risk for mortality among older Taiwanese sarcopenic patients. We applied a targeted metabolomic approach to study the plasma metabolites of adults aged ≥65 years, and identified the metabolic signature predictive of the mortality of sarcopenic patients who died within a 5.5-year follow-up period. Thirty-five sarcopenic patients who died within the follow-up period (*Dead* cohort) had shown a specific plasma metabolic signature, as compared with 54 patients who were alive (*Alive* cohort). Only 10 of 116 non-sarcopenic individuals died during the same period. After multivariable adjustment, we found that sex, hypertension, tetradecanoyl-carnitine (C14-carnitine), and docosahexaenoic acid (DHA)-containing phosphatidylcholine diacyl (PCaa) C38:6 and C40:6 were important risk factors for the mortality of sarcopenic patients. Low PCaa C38:6 levels and high C14-carnitine levels correlated with an increased mortality risk; this was even the same for those patients with hypertension (HTN). Our findings suggest that plasma PCaa C38:6 and acylcarnitine C14-carnitine, when combined, can be a better early biomarker for evaluating the mortality risk of sarcopenia patients.

## 1. Introduction

Sarcopenia is an age-related loss of lean muscle mass and a decline in muscle strength and performance [1]. It can result in frailty, falls, fracture, disability, hospitalization, decline in quality of life, and even mortality [2,3]. The prevalence of sarcopenia across different populations was estimated to range from 9.9% to 40.4% [4]. In Asian countries, it varied from 5.5% to 25.7%, with male predominance (5.1–21.0% in men vs. 4.1–16.3% in women) according to the Asian Working Group for Sarcopenia (AWGS) 2014 criteria [5,6]. With an update of the AWGS operational definition in 2019 [6], the prevalence of sarcopenia (21.3% in men and 13.8% in women) was even higher than that defined by the 2014 version (10.3% in men and 8.1% in women) in a Korean community [7]. As sarcopenia is relatively common among older adults, the medical costs and the provision of long-term care for the patients pose a burden on healthcare systems, making it an important health issue in the aged and hyper-aged societies.

Multiple risk factors, including age, lifestyle, physical activities, nutritional status, and co-morbidities (e.g., osteoporosis, cardiovascular diseases, etc.) are associated with sarcopenia [6,8,9] and its adverse outcomes [10]. Poor nutritional status can be an important underlying cause of sarcopenia. Low levels of serum protein [11,12], vitamin D [13,14] and ω-3 fatty acids [15] are associated with sarcopenia. The development of new methods or biomarkers for the early diagnosis of sarcopenia is critical to the well-being of patients [16,17]. Nutritional supplementation is recommended for the prevention and treatment of sarcopenia [18]. Dietary supplementation with ω-3 fatty acids, such as DHA and eicosapentaenoic acid (EPA), prevent muscle loss and its functional deterioration [19]. The relationship between these ω-3 fatty acids and the adverse outcomes (e.g., mortality) of sarcopenia remains unclear.

Three major types of ω-3 PUFAs, namely α-linolenic acid (ALA), DHA, and EPA, are physiologically relevant. ALA is mainly derived from plants, while DPA and EHA are enriched in deep-sea fish. Mammals cannot synthesize ALA and have to obtain it through diet. Although ALA can be converted to DPA and EHA, only small amounts of DPA and EHA are synthesized through hepatic conversion [20]. These ω-3 PUFAs are beneficial to neurological and visual development in fetuses [21], and are associated with a lower risk of obesity, metabolic syndrome, diabetes, cardiovascular diseases, arthritis, cognitive impairment, and neurodegenerative diseases [22].

Carnitine plays an important role in skeletal muscle energy metabolism [23], and is involved in the transport of fatty acids across the mitochondrial inner membrane for β-oxidation. The intracellular carnitine pool consists of carnitine and its short-, medium- and long-chain fatty acyl derivatives [24]. Increased levels of these carnitine derivatives are associated with elevated risk for heart failure [25], cardiovascular diseases [26], and type 2 diabetes [27]. Plasma acylcarnitines have also been shown to evaluate the prognosis of IgA nephropathy and the efficacy of treatment [28]. We have recently demonstrated that several acylcarnitines increased in the plasma of sarcopenic patients [29]. Elevated plasma levels of medium- and long-chain acylcarnitines were associated with the poor physical performance of the lower-extremities in older adults [30]. Nonetheless, the relationship between acylcarnitines and the mortality outcome of sarcopenia is obscure.

The identification of the disease-specific metabolic signature provides a better opportunity to assess the prognosis and clinical outcomes of patients. Previous metabolomic studies suggest that the distinct metabolic profiles of amino acids, lipids, and gut microbiome-related metabolites are related to muscle mass [31,32,33] and physical function [34,35,36,37,38,39]. Nonetheless, to the best of our knowledge, none have addressed whether such metabolic differences affect the mortality risk of sarcopenic patients.

Here, we apply metabolomic approaches to study the plasma metabolic profiles of sarcopenic patients and identify the plasma metabolic signature associated with the patients who died within the follow-up period. Statistical analyses reveal that the DHA-containing PC PCaa C38:6 is inversely associated with the mortality of sarcopenic patients. In contrast, C14-carnitine correlates with the mortality risk. We apply the receiver operating characteristic analysis to demonstrate the diagnostic accuracy of these metabolites. Sarcopenic patients are grouped according to their tertile levels of PCaa C38:6 and C14-carnitine, and to their hypertension (HTN) status. Subsequent analyses reveal that the sarcopenic patients with low plasma PCaa C38:6 levels and high C14-carnitine levels have an increased mortality. This also holds true for the sarcopenic patients with HTN. Thus, we demonstrate that these molecules can be used in combination to assess the mortality risk among sarcopenia patients.

## 2. Materials and Methods

### 2.1. Study Population and Study Design

The study was conducted according to the guidelines of the Declaration of Helsinki, and was approved on 1 April 2013 by the Institutional Review Board of Chang Gung Memorial Hospital (No.: 201300961B0). We recruited 234 older adults who were aged ≥65 years (mean age = 81.9 ± 7.2 years) and had received annual physical and health examinations at a retirement home in northern Taiwan from January 2014 to December 2014. Informed consent was obtained from all subjects involved in the study. Blood specimens were collected from the participants in fasting condition for analysis of hematological and biochemical parameters as described in Table 1, according to the standardized procedures of Chang Gung Memorial Hospital Department of Laboratory Medicine. Plasma obtained from the same blood specimens was retained for the metabolomic analyses. Muscle mass, muscle strength (handgrip strength; HGS), and performance (gait speed; GS) were examined. The follow-up data were also collected from annual physical and health examinations until 2019 or the last available visit.

The study flowchart is shown in Figure S1. Of all participants, 11 cancer patients were excluded. We defined sarcopenia according to AWGS 2019 criteria. Low HGS was defined as HGS < 28 kg for men and <18 kg for women. Low GS was defined as GS < 1 m/s in 4-m walk GS test. Appendicular skeletal muscle mass was measured by dual-energy X-ray absorptiometry (DXA). The appendicular skeletal muscle index (ASMI) is defined as skeletal lean muscle mass divided by the height squared (kg/m^2^). Low muscle mass was defined as ASMI < 7.0 kg/m^2^ for men and <5.4 kg/m^2^ for women. Forty-four participants with normal HGS and GS were classified as normal individuals. One hundred and seventy-nine participants with low HGS and/or low GS were further examined for their appendicular skeletal muscle mass. Ninety-eight participants with low muscle mass were classified as sarcopenic patients, while the rest with normal muscle mass but with poor physical function were considered as non-sarcopenic low physical function (LPF) subjects (*N* = 81). The normal and non-sarcopenic LPF subjects were grouped as non-sarcopenic cohort. After 5.5 years of follow-up, 106 non-sarcopenic subjects were alive, while only 10 of them deceased. During the same period, 54 sarcopenic patients were alive (group 3 *Alive* cohort); 9 patients were bedridden; and 35 patients deceased (group 4 *Dead* cohort). The bedridden patients were excluded from the analysis. After the initial physical and health examinations in 2014, the patients received health checkups until 2019 or the last available visit. The follow-up lasted for up to 5.5 years. The information on mortality, health condition, medical treatment, and hospitalization of participants was collected from electronic medical records.

### 2.2. Assessment of Sarcopenia and Muscle Mass and Physical Functions

The muscle mass was measured using dual energy X-ray absorptiometry (GE Lunar iDXATM; GE Healthcare, Madison, WI, USA), and ASMI was calculated in units of kilogram per meter squared. Low muscle mass was defined as an ASMI of less than 7.0 kg/m^2^ for men and less than 5.4 kg/m^2^ for women [6].

Muscle strength was assessed by hand grip strength (HGS) using a hand dynamometer (Jamar Plus^+^ Digital Hand Dynamometer; Sammons Preston, Bollingbrook, IL, USA). Low muscle strength was defined as grip strength < 28 kg or <18 kg for men and women, respectively [6].

Physical function or performance was assessed by gait speed (GS). Each participant was asked to walk a distance of 4 m in order to measure his or her GS. Low physical function was defined as a usual GS < 1.0 m/s for both men and women [6,40].

According to the Asian Working Group for Sarcopenia (AWGS) 2019 criteria, sarcopenia (S) was defined as the presence of low muscle mass, plus low muscle strength or low physical performance [6].

### 2.3. Targeted Metabolomic Analysis of Plasma

Metabolites in plasma samples were determined using a commercially available kit (AbsoluteIDQ p180; BIOCRATES Life Sciences AG, Innsbruck, Austria). Briefly, an aliquot of 10 μL plasma was prepared and processed according to the manufacturer’s instructions as previously described [41,42]. Biogenic amines were measured using the ultra-high performance liquid chromatography-tandem mass spectrometry (UPLC-MS/MS), and lipid species were quantified by flow injection analysis (FIA) coupled with MS/MS. Metabolite concentrations were calculated and expressed in μM. The global analysis of plasma metabolites was performed as described elsewhere [43].

### 2.4. Chemometric and Statistical Analyses

For global metabolomic analysis, partial least squares-discriminant analysis (PLS-DA was performed using MetaboAnalyst 5.0 online platform (https://www.metaboanalyst.ca/home.xhtml; accessed on 17 August 2023) [44]. Those variables (metabolites) with variable importance in projection (VIP) score > 3 in PLS-DA model were selected. The ANOVA with Tukey’s HSD test was performed using the same platform. The targeted metabolomics data obtained with AbsoluteIDQ p180 kit were processed and analyzed using MetIDQ software (Oxygen-DB110-3005).

All other statistical analyses were performed using SPSS software version 19.0 (SPSS, Chicago, IL, USA). The baseline characteristics and metabolite concentrations are presented as mean ± standard deviation for continuous variables, and as counts (or percentages) for categorical variables. The Student’s *t* test or Mann–Whitney test was used for a comparison of continuous variables between different groups where appropriate, and the chi-square (χ^2^) test was used for a comparison of categorical variables between different groups where appropriate. A *p*-value of <0.05 was considered statistically significant. A false discovery rate (FDR)-adjusted *p*-value (P_FDR_) of less than 0.05 was considered statistically significant.

A Cox proportional hazards regression model was applied to determine independent predictors of mortality after adjustment for covariates. Each statistical model contained age, sex, ASMI, GS, BMI, comorbidities, clinical and hematological parameters (RBC, hemoglobin, hematocrit, BUN, albumin, HDLC, cortisol, creatinine and uric acid), and the abundance of a specified metabolite (C14-carnitine (in nM) for both models 1 and 2; PCaa C38:6 (in μM) for model 1 and PCaa C40:6 (in μM) for model 2. Hazard ratio (HR) and 95% confidence interval (CI) were calculated. The survival analysis was performed using the Kaplan–Meier method. The log-rank test was applied where appropriate. The diagnostic accuracy of C14-carnitine and PCaa C38:6 was determined using receiver operating characteristic (ROC) analysis, and the area under curve (AUC) was used to estimate its performance.

## 3. Results

### 3.1. Demographics

The demographic, hematological, and biochemical characteristics of the study population are summarized in Table 1. Hematological and biochemical data were collected during the period from January 2014 to December 2014, while the demographic data were collected during this period and the subsequent follow-up period. Among 234 older adults (mean age = 81.9 ± 7.2 years) recruited, 223 participants were classified into 44 normal subjects (mean age = 76.5 ± 7.9 years), 81 non-sarcopenic LPF subjects (i.e., those with normal muscle mass have low handgrip strength (HGS) or low gait speed (GS)) (mean age = 81.3 ± 6.5 years), and sarcopenic patients (mean age = 84.7 ± 5.6 years) (Figure S1). The 5.5-year mortality of sarcopenic patients (35.7%) was significantly higher than those of normal (9.1%) and non-sarcopenic LPF subjects (7.4%) (Table S1). The normal and non-sarcopenic LPF subjects were categorized as the non-sarcopenic cohort.

It is interesting to identify any factors associated with the higher mortality of sarcopenic patients. For the non-sarcopenic cohort, after nine bedridden subjects were excluded, 106 were alive (i.e., group 1 *Alive* cohort) (mean age = 79.4 ± 7.3) and 10 died within the 5.5-year follow-up period (i.e., group 2 *Dead* cohort) (mean age = 81.2 ± 7.3). With the exclusion of nine bedridden sarcopenic patients, 54 patients who survived at the end of study (i.e., group 3 *Alive* cohort) (mean age = 82.8 ± 5.7) and 35 patients who died within the follow-up period (i.e., group 4 *Dead* cohort) (mean age = 87.8 ± 4.1) were subjected to further analyses. An analysis of their demographics and clinical features revealed significant differences (*p* < 0.01) in age, sex, sarcopenia-related parameters (appendicular skeletal muscle index (ASMI), and gait speed (GS)), hemoglobin, hematocrit, blood urea nitrogen (BUN), albumin, creatinine, and uric acid between the sarcopenic *Alive* and *Dead* patient cohorts (Table 1). These parameters did not differ significantly between the non-sarcopenic *Alive* and *Dead* patient cohorts at the same significance level.

### 3.2. Metabolomic Analysis of the Plasma of Sarcopenic Patients

For the identification of a metabolic signature that is predictive of the mortality of sarcopenic patients, the plasma specimens collected from non-sarcopenic subjects and sarcopenic patients were subjected to the untargeted analyses of lipophilic metabolites (Figure S2). There were 3276 unique features identified in these datasets. We further analyzed the datasets using the partial least squares discriminant analysis (PLS-DA). Fifty-three metabolites with a variable importance in the projection (VIP) score greater than 3 were selected. In addition, thirty-nine metabolites showed significant differences in abundance after the datasets were independently analyzed using ANOVA with Tukey’s Honest Significant Differences (HSD) test. These two sets of the selected metabolites had four members in common, and were found to be the [M+H] and [M+Na] adducts of PCaa C38:6 and PCaa C40:6 (Figure S2).

For better quantitative results, we resorted to a targeted approach and used AbsoluteIDQ p180 to analyze all the specimens. We specifically focused on the targeted metabolic profiles of sarcopenic patients. The metabolites whose abundances differed between the *Alive* (i.e., group 3) and *Dead* (i.e., group 4) cohorts of sarcopenic patients were selected (*p* < 0.05, Table S2). Within this metabolite list, those associated with the false discovery rate (FDR)-adjusted *p* values (P_FDR_) < 0.05 were considered significant and are shown in Table 2. Of these plasma metabolites, PCaa C38:6, PCaa C40:6, PCae C38:0, PCae C40:1, SM (OH) 22:1, and SM (OH) 22:2 were lower in abundance in the *Dead* cohort than the *Alive* cohort. The others, including C14-carnitine, C3-DC (C4-OH)-carnitine, and symmetric dimethylarginine (SDMA), showed the opposite trend. In contrast, there were no significant differences in the abundances of these metabolites between the *Alive* and *Dead* cohorts of non-sarcopenic patients.

### 3.3. Plasma Metabolites Associated with the Mortality of Sarcopenic Patients

The Cox proportional hazards regression model was applied to determine which metabolites are associated with the risk for the all-cause mortality of sarcopenic patients. After adjustment for the covariates including age, sex, and comorbidities (i.e., HTN, diabetes, hyperlipidemia, coronary artery disease (CAD), stroke, chronic kidney disease (CKD), chronic obstructive pulmonary disease (COPD), and osteoporosis), other physical and clinical parameters with significant differences (*p* < 0.05) (i.e., ASMI, GS, BMI, RBC, hemoglobin, hematocrit, BUN, albumin, HDLC, creatinine, cortisol and uric acid), and significantly changed metabolites, the analyses revealed that sex, HTN, and C14-carnitine were associated with an increased risk of mortality (*p* < 0.05). The two PC species, which included PCaa C38:6 [model 1; HR = 0.962, 95% CI = 0.929–0.996] and PCaa C40:6 [model 2; hazard ratio (HR) = 0.875, 95% CI = 0.789–0.971], were associated with a decreased risk of mortality (*p* < 0.05) (Table 3).

The PCs with formulae PCaa C38:6 and PCaa C40:6 were subjected to MS/MS analysis. The most representative species of PCaa C38:6 and PCaa C40:6 molecules were PCaa (16:0/22:6) and PCaa (18:0/22:6), respectively (Figure S3). All of them contain DHA as their fatty acyl constituent. Our findings suggest that specific DHA-containing PCs are related to the risk for the sarcopenia-associated all-cause mortality.

### 3.4. C14-Carnitine and PCaa C38:6 as Predictor of Mortality

**Figure 1:**
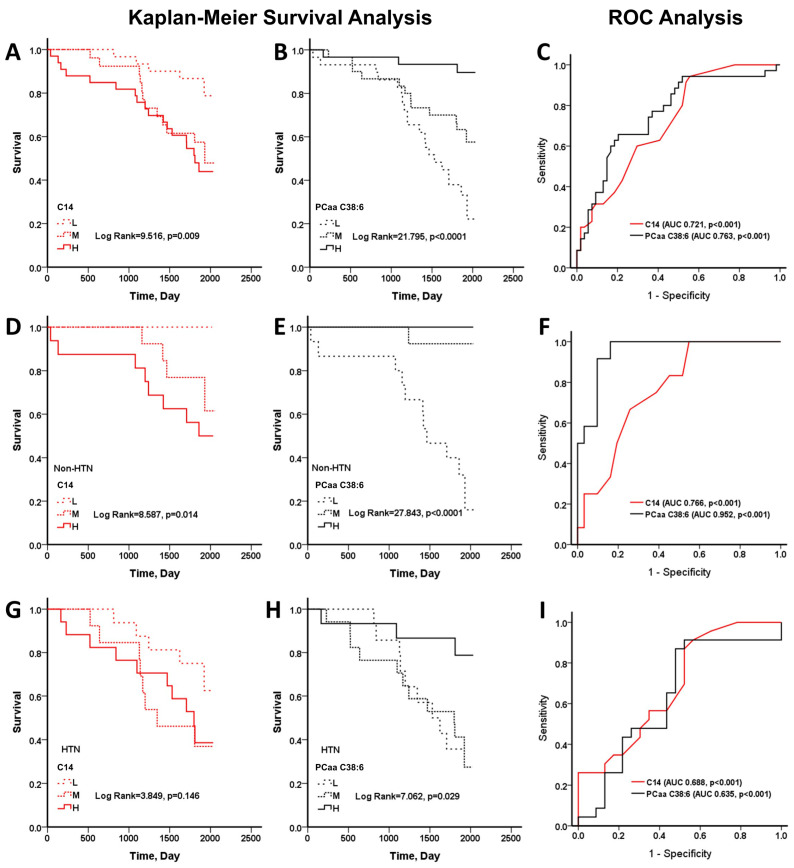
The Kaplan–Meier survival curves of the sarcopenic patients with different plasma levels of C14-carnitine and PCaa C38:6. The sarcopenic patients were categorized into groups according to the tertiles of plasma C14-carnitine (*C14*) and PCaa C38:6 levels. The survival curves of the patients with low (L), mid (M), and high (H) plasma levels of C14-carnitine and PCaa C38:6 are shown in (**A**,**B**), respectively. The sarcopenic patients with different plasma C14-carnitine (**D**,**G**) or PCaa C38:6 (**E**,**H**) levels were further classified according to the absence (non-HTN) and presence (HTN) of hypertension. The survival curves for the non-HTN (**D**,**E**) and HTN (**G**,**H**) groups with L, M and H plasma C14-carnitine and PCaa C38:6 levels are shown. (**C**,**F**,**I**) The receiver operating characteristic (ROC) curves of both C14-carnitine (*C14*) and PCaa C38:6 for the all-cause mortality of sarcopenic patients (**C**), and non-HTN (**F**) and HTN (**I**) cohorts are shown.

HTN has been reported to be associated with sarcopenia [45], and with an increased risk for mortality (Table 3). The Kaplan–Meier survival analysis also revealed a higher mortality of sarcopenic patients in the HTN cohort (Figure S4D) than in the non-HTN cohort (Figure S4C). It is speculative whether HTN affects the efficacy of C14-carnitine or DHA-containing PCs as predictors for all-cause mortality. As PCaa C38:6 is at higher concentration than PCaa C40:6 in plasma, we focused on the analysis of PCaa C38:6 and C14-carnitine. The plasma concentrations of PCaa C38:6 and C14-carnitine were divided into tertiles T1 (low, L), T2 (mid, M), and T3 (high, H) (with the means of these concentration tertile groups shown in Table S3), and the sarcopenic patients were grouped accordingly. The mean values of PCaa C38:6 in T1, T2 and T3 groups were 53.92 ± 8.13, 75.48 ± 4.88, and 103.70 ± 18.55 μM, respectively; those of C14-carnitine in T1, T2, and T3 groups were 30.37 ± 2.36, 36.35 ± 1.52, and 43.82 ± 4.90 nM, respectively (Table S3). The high (H) and mid- (M) C14-carnitine levels were associated with substantial decreases in survival (Figure 1A). The low (L) and mid (M) PCaa C38:6 levels were associated with substantial decreases in survival (Figure 1B). The diagnostic accuracy of C14-carnitine (area under curve (AUC) = 0.721, *p* < 0.001) and PCaa C38:6 (AUC = 0.763, *p* < 0.001) (Figure 1C) in terms of all-cause mortality was good.

To test whether HTN affects the diagnostic accuracy of PCaa C38:6, we further divided sarcopenic patients into non-HTN and HTN cohorts. For the non-HTN cohort, only low C14-carnitine was associated with a significant increase in survival (Figure 1D), while low PCaa C38:6 level showed the opposite trend (Figure 1E). The diagnostic accuracy of both C14-carnitine (AUC = 0.766, *p* < 0.001) and PCaa C38:6 (AUC = 0.952, *p* < 0.001) for assessing mortality was even higher in the non-HTN cohort, as compared with that of the entire sarcopenic patient population (Figure 1F vs. Figure 1C). For the HTN cohort, high (H)-PCaa C38:6 levels were associated with significant increases in survival (Figure 1H). The diagnostic accuracy of C14-carnitine (AUC = 0.686, *p* < 0.01) and PCaa C38:6 (AUC = 0.635, *p* < 0.01) was lower for the HTN cohort than for the non-HTN cohort (Figure 1I). These findings suggest that high plasma PCaa C38:6 levels are associated with the better survival of sarcopenic patients, even those with HTN.

**Figure 2:**
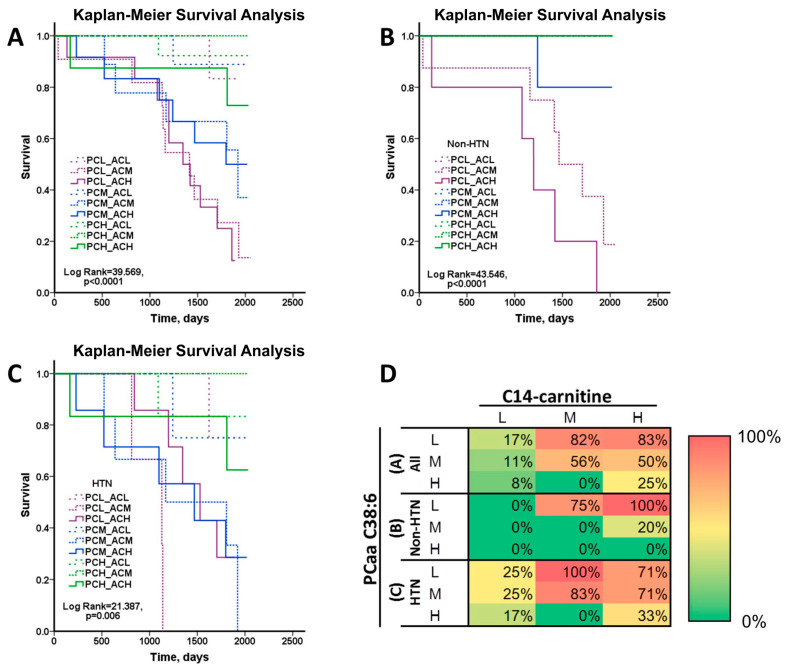
The combinations of different plasma C14-carnitine and PCaa C38:6 levels are associated with survival among all, non-hypertension, and hypertension sarcopenic patients. The Kaplan–Meier plot comparing the overall survival of all sarcopenic patients (**A**), sarcopenic patients without hypertension (Non-HTN) (**B**), sarcopenic patients with hypertension (HTN) (**C**). The heatmap of mortality for C14-carnitine and PCaa C38:6 combinations indicates the percentages of death of all, non-HTN and HTN sarcopenic patients according to the L, M, and H tertile levels of PCaa C38:6 and C14-carnitine (**D**). The redder the color, the higher the mortality; the greener the color, the lower the mortality. The abbreviations for different C14-carnitine and PCaa C38:6 combinations are as follows: PCL_ACL, low PCaa C38:6 and low C14-carnitine levels; PCL_ACM, low PCaa C38:6 and mid-C14-carnitine levels; PCL_ACH, low PCaa C38:6 and high C14-carnitine levels; PCM_ACL, mid-PCaa C38:6 and low C14-carnitine levels; PCM_ACM, mid-PCaa C38:6 and mid-C14-carnitine levels; PCM_ACH, mid-PCaa C38:6 and high C14-carnitine levels; PCH_ACL, high PCaa C38:6 and low C14-carnitine levels; PCH_ACM, high PCaa C38:6 and mid-C14-carnitine levels; PCH_ACH, high PCaa C38:6 and high C14-carnitine levels.

As both C14-carnitine and PCaa C38:6 are important predictors for mortality in sarcopenic patients, we examined the possibility that a combination of these two metabolites can be a more adequate biomarker. The study populations were categorized into nine groups according to tertile C14-carnitine and PCaa C38:6 (Table S3). The Kaplan–Meier plots presented all sarcopenia patients (Figure 2A) and the non-HTN (Figure 2B) and HTN (Figure 2C) patients within this cohort. The mortalities of various cohorts were tabulated in the heatmap of mortality (Figure 2D). For the non-HTN sarcopenia patients, death occurred only in the low PCaa C38:6, mid C14-carnitine (PCL_ACM) cohort, the low PCaa C38:6, high C14-carnitine (PCL_ACH) cohort, and the mid PCaa C38:6, high C14-carnitine (PCM_ACH) cohort (Figure 2B,D). For the HTN sarcopenia patients, the mortalities increased significantly in the low PCaa C38:6, mid C14-carnitine (PCL_ACM) cohort, the mid PCaa C38:6, mid C14-carnitine (PCM_ACM) cohort, the low PCaa C38:6, high C14-carnitine (PCL_ACH) cohort, and the mid PCaa C38:6 and high C14-carnitine (PCM_ACH) cohort (Figure 2C,D). A high plasma PCaa C38:6 level is indicative of the better survival of sarcopenia patients. Even so, the mortality of the HTN patients with high plasma PCaa C38:6 levels increased moderately when they had high C14-carnitine levels (i.e., high PCaa C38:6, high C14-carnitine (PCH_ACH) HTN cohort). These findings suggest that the low plasma PCaa 38:6 and high plasma C14-carnitine are associated with the mortality of sarcopenic patients. There appears to be an interaction between these factors in determining the mortality outcome.

## 4. Discussion

In the present study, we assessed the mortality of sarcopenic patients 5.5 years after the initial health checkup. We identified the plasma metabolic signature associated with the mortality of sarcopenic patients. Of the identified metabolites, the low plasma PCaa C38:6 level and high plasma C14-carnitine level are associated with an increased risk of mortality among sarcopenic patients.

Carnitine serves to transport the fatty acids as acylcarnitines into mitochondria for β-oxidation. Mitochondrial activity declines during aging [46,47], resulting in an inefficient fatty acid β-oxidation in various tissues including skeletal muscle. It is conceivable that intracellular acylcarnitine accumulation leads to its efflux and elevation of plasma acylcarnitine level. Plasma acylcarnitines can be used as a marker of impaired mitochondrial energy metabolism, and are indicative of mitochondrial dysfunction [48,49]. Consistent with the deleterious changes in mitochondria during aging, the long-chain and very long-chain acylcarnitines increased with the age of healthy individuals [50]. Abnormal mitochondrial metabolism is implicated in disease pathogenesis. An increase in plasma acylcarnitines has been associated with an elevated risk for diseases such as heart failure [25], cardiovascular diseases [26], and type 2 diabetes [27]. The elevation of the levels of acylcarnitines, such as C14-carnitine, may be related to skeletal mitochondrial dysfunction and pathogenesis of sarcopenia. We have recently reported that the plasma carnitines butyrylcarnitine (C4), hexanoylcarnitine (C6), and 3-hydroxyoleoylcarnitine (C18:1-OH) significantly increase in sarcopenic patients [29]. It is wondered why acylcarnitines may be associated with the mortality risk of sarcopenia. Long-chain acylcarnitines can elicit cell stress and cell death responses in a model of skeletal muscle myotubes [51]. Additionally, acylcarnitines may stimulate the pro-inflammatory signaling pathways in macrophages. The C14-carnitine treatment results in MyD88-mediated induction of pro-inflammatory cytokines, probably via JNK and ERK activation [52]. Chronic, low-grade inflammation or “inflammaging” occurs with aging and contributes to the pathogenesis of chronic degenerative diseases [53], including sarcopenia [54]. High plasma C-14 carnitine may cause systemic inflammation, and together with mitochondrial dysfunction, increases the mortality risk of the sarcopenic patients.

There has been an interest in studying the relationship between ω-3 polyunsaturated fatty acids (PUFAs) and health outcomes [55]. Several studies have suggested an association between ω-3 PUFAs and muscle health. Dietary intake of ω-3 PUFAs enhances skeletal muscle buildup and its function [56,57]. Reduced ω-3 PUFA intake is associated with sarcopenia in elderly patients with type 2 diabetes [58]. Several mechanisms underlying the potential protective effect of ω-3 PUFAs have been proposed. Chronic inflammation and the age-related expression of pro-inflammatory cytokines may cause muscle wasting [59]. The ω-3 PUFAs have anti-inflammatory and antioxidative activities that counteract the deleterious effect of cytokines [60]. For instance, DHA is known to suppress acylcarnitine-induced expression of pro-inflammatory cytokines in macrophages [52]. The ω-3 fatty acids can be converted to specialized proresolving mediators (SPM), which blocks the transition of acute inflammation to a chronic response and promotes tissue repair [61]. It is likely that these molecular processes mitigate the muscle wasting process and other low-grade inflammation. Additionally, ω-3 PUFAs also stimulate satellite cells for the regeneration of damaged muscle and muscle cell differentiation [62,63]. They may improve the muscle strength and functions through the enhancement of neuromuscular junction conductivity and muscle contractile capacity [64]. The SPM resolvin D1 is also known to regulate muscle stem cells and to enhance skeletal myofiber regeneration [65]. At the molecular level, these PUFAs may modulate cellular pathways and affect the muscle cell physiology. They can activate the mTOR pathway and enhance p70S6K and FOXA3 phosphorylation to promote protein synthesis [66,67]. DHA may inhibit the proteasome and lysosomal degradation of muscle proteins [68]. Apart from the beneficial effect on skeletal muscles, ω-3 fatty acids have other health-promoting effects, such as cardioprotective, antiarrhythmic, and antithrombotic effects [69]. They stabilize atherosclerotic plaques and reduce the risk of cardiovascular events [70]. Additionally, the ω-3 PUFAs have a neuroprotective effect [71]. DHA promotes neurite outgrowth, synaptogenesis, and neural stem cell differentiation. The lowering of plasma ω-3 PUFAs levels in sarcopenic patients may diminish anti-inflammatory capacity and health-promoting effects, causing the deterioration of physiological functions; this is likely to increase the mortality risk.

An association between HTN and sarcopenia has been reported [45]. Dietary intake of the PUFA-enriched food is beneficial for the health of HTN patients [72] and those with cardiovascular diseases [73]. Consistent with such nutritional findings, the survival of HTN patients with high plasma PCaa C38:6 levels is higher than those with mid or low levels. From the viewpoint of diagnosis, the presence of HTN diminishes the diagnostic accuracy of PCaa C38:6 in predicting sarcopenia-associated mortality. Though the AUC of the receiver operating characteristic (ROC) curve for PCaa C38:6 was 0.635, it was still useful as a predictor for mortality among the sarcopenic HTN patients.

It has been reported that the chronic kidney disease (CKD) patients are susceptible to the development of sarcopenia [74]. It is speculative whether CDK promotes the death of sarcopenic patients. We observed an increase in the percentage of CKD patients in the sarcopenia cohort as compared to that of the non-sarcopenia cohort. Despite this, there was no significant difference in the percentage of CKD patients between the *Alive* and *Dead* cohorts of sarcopenic patients (Table 1). It is unlikely that CKD contributes to the death of these patients. As regards the molecular risk factors, C14-carnitine (nM) and PCaa C38:6 (μM) (or PCaa C40:6, μM) are associated with the risk for mortality among sarcopenic patients after adjustment for comorbidities that include CKD (Table 3). Likewise, these molecules are associated with the risk for mortality among sarcopenic patients after adjustment for age. Hence, the presence of CKD and age do not call into question the validity of the conclusion about these molecules.

**Figure 3:**
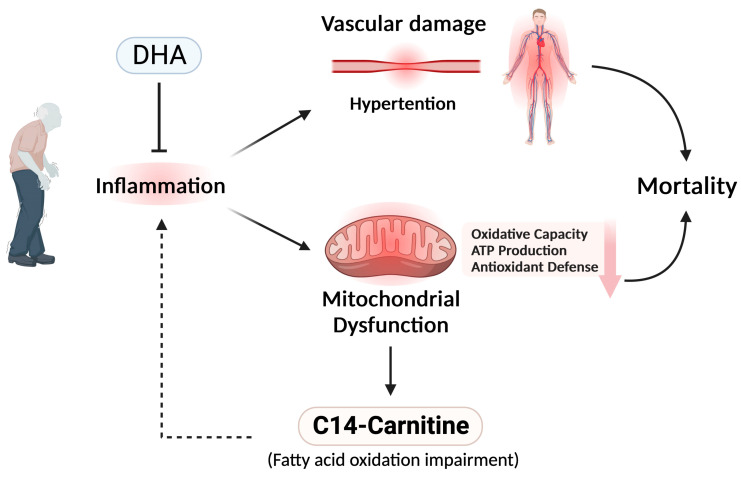
A schematic diagram showing the probable roles of C14-carnitine and DHA released from PCaa in the determination of the most adverse outcome of sarcopenia mortality. Please refer to the Discussion for explanation.

The combined metabolomic and epidemiological approach taken by our study has an implication about the pathogenic processes leading to the demise of sarcopenic patients. It is hypothesized that mitochondrial dysfunction occurs during aging. The release of C14-carnitine into the bloodstream may promote chronic systemic low-grade inflammation (inflammaging) [75] and aggravate the vascular inflammation and dysfunction associated with hypertension [76]. Mitochondrial dysfunction is accompanied by a reduction in oxidative phosphorylation capacity, ATP production, and antioxidant defense. These biochemical events exacerbate the pathophysiological condition. DHA released from PCaa exerts anti-inflammatory and antioxidative effects to contain the inflammation-induced damages. Moreover, DHA has other health-promoting functions (e.g., cardioprotective, antiarrhythmic, and antithrombotic activities, and a tissue repair-promoting effect). The reduction in DHA-containing phospholipids and the increase in C14-carnitine contribute to systemic inflammation, and predispose the individuals to serious health deterioration and even death. The model is illustrated in Figure 3.

This study had several limitations. We did not monitor the change in ω-3 PUFA-containing phospholipid content during the follow-up period. The retirement home dwellers who died within the follow-up period did so due to natural causes and were not linked to any hospital (discharge) records. Nor did they receive any post-mortem examination. Moreover, it is preferable to increase the number of participants in the study.

The proposed PCaa C38:6/C14-carnitine biomarker panel may find its way into clinical application. The utilization of such biomarkers provides an opportunity for timely lifesaving therapeutic intervention for sarcopenic patients, and is beneficial for their well-being. This also raises the possibility of nutritional supplementation to promote the health of sarcopenic patients.

## 5. Conclusions

There is a plasma metabolic signature associated with sarcopenic patients who died within the follow-up period. Low plasma levels of DHA-containing phosphatidylcholine PCaa C38:6 and high plasma levels of acylcarnitine C14-carnitine are associated with an increased mortality in the sarcopenia cohort. These metabolites can be used as potential biomarkers for evaluating the mortality risk among sarcopenic patients.

## Figures and Tables

**Table 1:** Demographics of the alive and dead cohorts of non-sarcopenic subjects and sarcopenic patients, and their blood, biochemical, and clinical parameters.

	Non-Sarcopenic			Sarcopenic		
Parameter	Alive, *N* = 106	Dead, *N* = 10	*p*-Value ^1^	Alive, *N* = 54	Dead, *N* = 35	*p*-Value ^1^
***Age & body index, and physical function***						
Age (years)	79.4 ± 7.3	81.2 ± 7.3	0.4603	82.8 ± 5.7	87.8 ± 4.1	<0.0001
Sex (% male)	25%	40%	0.4537	44%	89%	<0.0001
ASMI (kg/m^2^)	6.2 ± 0.8	6.7 ± 0.6	0.0491	5.5 ± 0.9	6.0 ± 0.7	0.0053
HGS (kg)	19.0 ± 7.5	21.8 ± 8.9	0.2683	17.7 ± 7.1	18.5 ± 4.7	0.5356
GS (m/s)	1.1 ± 0.3	0.9 ± 0.3	0.0413	1.1 ± 0.3	0.8 ± 0.3	<0.0001
BMI (kg/m^2^)	24.2 ± 3.0	26.5 ± 3.7	0.0270	21.2 ± 2.5	22.7 ± 3.1	0.0152
***Complete blood count***						
WBC (10^3^/μL)	5.5 ± 1.5	5.6 ± 1.1	0.7260	5.5 ± 1.3	5.7 ± 1.3	0.3403
Segment (%)	56.5 ± 8.8	57.9 ± 10.1	0.6231	58.5 ± 8.1	59.8 ± 10.4	0.5110
Lymphocyte (%)	33.9 ± 8.4	33.0 ± 9.7	0.7259	31.6 ± 7.2	29.6 ± 9.5	0.2709
Monocyte (%)	6.2 ± 1.6	5.4 ± 1.1	0.1637	6.4 ± 1.5	5.9 ± 1.4	0.1161
Eosinophil (%)	2.9 ± 2.1	3.3 ± 2.0	0.5753	3.0 ± 2.5	4.1 ± 3.6	0.1215
Basophil (%)	0.6 ± 0.4	0.4 ± 0.2	0.0813	0.6 ± 0.4	0.5 ± 0.3	0.7907
RBC (10^6^/μL)	4.3 ± 0.5	4.7 ± 0.5	0.0558	4.3 ± 0.4	4.1 ± 0.5	0.0362
Hemoglobin (g/dL)	13.1 ± 1.6	13.7 ± 0.9	0.2337	13.3 ± 1.3	12.4 ± 1.8	0.0097
Hematocrit (%)	39.5 ± 4.2	40.8 ± 2.2	0.1087	39.8 ± 3.6	37.2 ± 4.7	0.0049
MCV (fL)	91.3 ± 5.8	88.4 ± 8.8	0.3349	92.1 ± 3.8	90.5 ± 5.2	0.1237
MCH (pg/cell)	30.4 ± 2.4	29.7 ± 3.3	0.4540	30.8 ± 1.3	30.0 ± 2.6	0.1267
MCHC (gHb/dL)	33.2 ± 1.0	33.6 ± 0.7	0.2672	33.4 ± 0.7	33.1 ± 1.5	0.2942
RDW (%)	13.6 ± 1.5	14.1 ± 1.9	0.3019	13.4 ± 0.6	13.9 ± 1.6	0.0873
Platelets (10^3^/μL)	203.6 ± 49.0	176.7 ± 43.2	0.0962	193.4 ± 49.8	192.9 ± 45.6	0.9647
***Biochemical test***						
Cholesterol (mg/dL)	184.2 ± 36.7	185.1 ± 55.7	0.9614	180.1 ± 31.8	168.3 ± 38.1	0.1180
Triglyceride (mg/dL)	109.5 ± 58.0	96.6 ± 45.9	0.4971	88.2 ± 33.7	88.3 ± 51.7	0.9927
LDLC (mg/dL)	108.3 ± 32.6	111.9 ± 45.2	0.7471	104.5 ± 26.0	100.1 ± 31.1	0.4647
HDLC (mg/dL)	53.3 ± 11.9	53.9 ± 10.0	0.8799	57.4 ± 14.3	49.7 ± 12.2	0.0104
Insulin (μlU/mL)	6.7 ± 8.6	6.1 ± 3.7	0.6510	4.9 ± 7.5	4.7 ± 3.7	0.8697
Glucose (mg/dL)	97.2 ± 15.4	100.2 ± 15.8	0.5540	97.6 ± 22.2	101.3 ± 22.8	0.4611
HbA1c (%)	5.8 ± 0.5	5.9 ± 0.6	0.8402	5.9 ± 0.7	5.9 ± 0.8	0.7032
HOMA-IR	1.7 ± 2.1	1.6 ± 1.3	0.8948	1.2 ± 1.7	1.2 ± 1.0	0.9367
Albumin (g/dL)	4.4 ± 0.3	4.2 ± 0.2	0.0561	4.4 ± 0.2	4.2 ± 0.3	0.0008
Total protein (g/dL)	7.0 ± 0.4	6.9 ± 0.3	0.4134	7.0 ± 0.4	6.9 ± 0.4	0.1557
BUN (mg/dL)	17.4 ± 8.8	15.0 ± 2.3	0.0358	16.3 ± 4.3	24.8 ± 12.4	0.0004
Creatinine (mg/dL)	0.9 ± 0.9	0.8 ± 0.2	0.5016	0.8 ± 0.3	1.3 ± 0.9	0.0024
AST/GOT (U/L)	26.9 ± 9.9	32.4 ± 14.3	0.1117	28.7 ± 8.9	25.1 ± 8.4	0.0567
ALT/GPT (U/L)	20.3 ± 18.9	25.0 ± 9.9	0.2163	19.7 ± 12.7	16.5 ± 8.6	0.1534
ALKP (U/L)	67.4 ± 18.7	72.9 ± 35.0	0.6356	59.6 ± 15.5	64.7 ± 24.3	0.2718
Total bilirubin (mg/dL)	0.7 ± 0.3	0.7 ± 0.2	0.9267	0.8 ± 0.5	0.8 ± 0.5	0.8682
TSH (μIU/mL)	2.7 ± 2.2	2.6 ± 2.5	0.9643	2.4 ± 2.2	2.6 ± 1.8	0.6058
Uric acid (mg/dL)	5.5 ± 1.4	5.9 ± 2.2	0.6608	5.5 ± 1.2	6.7 ± 2.1	0.0018
T4 (μg/dL)	8.1 ± 1.7	9.0 ± 1.5	0.1570	8.2 ± 1.5	7.9 ± 1.9	0.3987
Cortisol (μg/dL)	13.6 ± 4.6	10.2 ± 5.4	0.0292	14.0 ± 4.5	16.3 ± 5.5	0.0352
Vitamin B12 (pg/mL)	762.4 ± 578.8	729.4 ± 361.6	0.8601	758.2 ± 632.6	789.8 ± 500.8	0.8039
***Comorbidities (%)***						
HTN	58%	60%	1.0000	43%	66%	0.0501
Diabetes	21%	30%	0.4472	31%	17%	0.1466
Hyperlipidemia	38%	40%	1.0000	31%	23%	0.4716
CAD	8%	0%	1.0000	9%	11%	0.7341
Stroke	4%	10%	0.3682	6%	17%	0.1462
CKD	8%	10%	1.0000	13%	29%	0.0971
COPD	15%	10%	1.0000	31%	37%	0.6490
Osteoporosis	26%	20%	1.0000	37%	34%	0.8249

Values are mean ± SD or N (%). ^1^ *Alive* vs. *Dead* cohorts of the same subject cohort. ALKP: alkaline phosphatase; ASMI: appendicular skeletal muscle mass index; ALT: alanine aminotransferase; AST: aspartate aminotransferase; BMI: body mass index; BUN: blood urea nitrogen; CAD: coronary artery disease; CKD: chronic kidney disease; COPD: chronic obstructive pulmonary disease; GOT: glutamate oxaloacetate transaminase; GPT: glutamate pyruvate transaminase; GS: gait speed; HDLC: High-density lipoprotein cholesterol; HbA1c: hemoglobin A1c; HGS: hand grip strength; HOMA-IR: homeostatic model assessment of insulin resistance; HTN: hypertension; LDLC: low-density lipoprotein cholesterol; MCH: mean corpuscular hemoglobin; MCHC: mean corpuscular hemoglobin concentration; MCV: mean corpuscular volume; RBC: red blood cell count; RDW: red cell distribution width; T4: 3,5,3′,5′-L-tetraiodothyronine; T-bilirubin: Total bilirubin; TSH: thyroid-stimulating hormone or thyrotropin; WBC: white blood cell count.

**Table 2:** Levels of differentially abundant metabolites in the plasma of the alive and dead cohorts of non-sarcopenic subjects and sarcopenic patients.

	Non-Sarcopenic			Sarcopenic			
Metabolite, μM	Alive, *N* = 106	Dead, *N* = 10	*p*-Value ^1^	Alive, *N* = 54	Dead, *N* = 35	*p*-Value ^1^	P_FDR_
PCaa C40:6	32.839 ± 9.150	33.620 ± 14.030	0.8664	30.774 ± 7.525	23.278 ± 7.337	1.2 × 10^−5^	0.0022
PCaa C38:6	88.734 ± 23.968	92.170 ± 32.817	0.6759	85.576 ± 22.647	66.229 ± 20.437	9.7 × 10^−5^	0.0058
C14	0.035 ± 0.006	0.036 ± 0.003	0.5532	0.035 ± 0.006	0.040 ± 0.006	0.0002	0.0072
C3-DC (C4-OH)	0.062 ± 0.027	0.061 ± 0.016	0.9229	0.058 ± 0.018	0.073 ± 0.023	0.0005	0.0140
PCae C38:0	1.754 ± 0.678	1.732 ± 0.728	0.9230	1.688 ± 0.540	1.298 ± 0.530	0.0012	0.0266
PCae C40:1	1.030 ± 0.280	0.986 ± 0.294	0.6317	1.073 ± 0.283	0.833 ± 0.246	8.9 × 10^−5^	0.0080
SM (OH) 22:1	49.325 ± 10.752	51.520 ± 13.016	0.5456	49.841 ± 9.998	41.783 ± 8.431	0.0002	0.0058
SM (OH) 22:2	52.066 ± 12.564	55.070 ± 11.672	0.4689	52.750 ± 9.190	44.994 ± 10.906	0.0005	0.0153
SDMA	0.760 ± 0.417	0.799 ± 0.180	0.5866	0.789 ± 0.257	1.120 ± 0.538	0.0014	0.0288

Values are mean ± SD. ^1^ *Alive* vs. *Dead* cohorts of the same subject cohort. C3-DC (C4-OH): malonylcarnitine (hydroxybutyrylcarnitine), C3-DC (C4-OH)-carnitine; C14: tetradecanoylcarnitine, C14-carnitine; SDMA: symmetric dimethylarginine; PCaa: phosphatidylcholine (diacyl; both fatty acids are linked via ester bonds); PCae: phosphatidylcholine (a fatty acid is linked via an ester bond, while another is linked via an ether bond); SM (OH): hydroxysphingomyelin.

**Table 3:** The Cox regression model with hazard ratios (HRs) and 95% confidence intervals (CIs) of the mortality associated with various factors among sarcopenic patients.

	Model 1		Model 2	
Variables	HR (95% CI)	*p* Value	HR (95% CI)	*p* Value
Sex	16.635 (2.449–113.005)	0.004	12.132 (1.663–88.494)	0.014
HTN	5.314 (1.639–17.228)	0.005	6.194 (1.813–21.189)	0.004
C14, nM	1.099 (1.015–1.190)	0.020	1.097 (1.013–1.186)	0.022
PCaa C38:6, μM ^1^	0.962 (0.929–0.996)	0.030		
PCaa C40:6, μM ^2^			0.875 (0.789–0.971)	0.012

Adjusted for factors including age (years), sex (female or male), comorbidities (absence or presence of HTN, diabetes, hyperlipidemia, CAD, stroke, CKD, COPD, or osteoporosis), ASMI (kg/m^2^), GS (m/s), BMI (kg/m^2^), RBC (10^6^/μL), hemoglobin (g/dL), hematocrit (%), HDLC (mg/dL), albumin (g/dL), BUN (mg/dL), creatinine (mg/dL), uric acid (mg/dL), cortisol (μg/dL), C14 (C14-carnitine; nM), and ^1^ PCaa C38:6 (μM) or ^2^ PCaa C40:6 (μM).

## Data Availability

The data presented in this study are available upon request. The data are not publicly available for protection of detailed patient data.

## Supplementary Materials

The following supporting information can be downloaded at: https://www.mdpi.com/article/10.3390/nu16050611/s1, Figure S1: The study flow diagram. Two hundred and thirty-four participants were enrolled in the present study, and plasma samples were collected. Of these participants, 11 cancer patients were excluded. Forty-four normal subjects, 81 non-sarcopenic LPF subjects, and 98 sarcopenic patients were categorized according to the AWGS 2019 criteria. The normal and non-sarcopenic LPF subjects were grouped as non-sarcopenic subjects. Plasma samples were subjected to metabolomic analyses. All subjects were followed up for 5.5 years. One hundred and six non-sarcopenic subjects were (group 1) alive, and 10 (group 2) deceased. Fifty-four sarcopenic patients (group 3) were alive, and 35 (group 4) died; Figure S2: Global analysis of plasma metabolites in sarcopenic and non-sarcopenic patients. Plasma collected from the non-sarcopenia alive (group 1), non-sarcopenia dead (group 2), sarcopenia alive (group 3) and sarcopenia dead (group 4) patient cohorts (as defined in Figure S1) were subjected to global metabolomic analysis. A total of 3276 unique features were identified. The datasets were analyzed using PLS-DA. Fifty-three metabolites had VIP scores greater than 3. The datasets were independently analyzed using ANOVA with Tukey’s HSD test. Thirty-nine differentially abundant metabolites were found (*p* < 0.05). The Venn diagram shows that only four metabolites fit both criteria. These metabolites were identified to be the [M+H] and [M+Na] adducts of PCaa C38:6 and PCaa C40:6; Figure S3: The proportion of different molecular species with formulae PCaa C38:6 and PCaa C40:6. The plasma samples were analyzed using TOF-MS/MS for identification and quantification of the molecular species with respective chemical formulae. The proportion was calculated accordingly. Data are mean ± SD, *N* = 86; Figure S4: The Kaplan–Meier survival curves of the non-sarcopenic (*N*) or sarcopenic (*S*) patients without (*Non-HTN*) or with hypertension (*HTN*). (A,B) All participants were categorized into N and S cohorts (A), or non-HTN and HTN cohorts (B). (C,D) All participants were categorized into non-HTN (C) or HTN (D) cohorts, each of which was further subdivided into N and S cohorts; Table S1: The 5.5-year mortality of all participants; Table S2: Differentially abundant metabolites in the plasma of the alive and dead cohorts of non-sarcopenic subjects and sarcopenic patients; Table S3: The mean plasma concentrations of PCaa C38:6 and C14-carnitine and the mortality of sarcopenic patients grouped on the basis of tertile concentrations and hypertension status.
