# Supplementary material for: Combined Plasma DHA-Containing Phosphatidylcholine PCaa C38:6 and Tetradecanoyl-Carnitine as an Early Biomarker for Assessing the Mortality Risk among Sarcopenic Patients
Source: Nutrients. 2024 Feb 23;16(5):611. doi: 10.3390/nu16050611 (PMC10933763; doi:10.3390/nu16050611)
Supplement: Supplementary file 1 [file nutrients-16-00611-s001.zip › nutrients-2840737-supplementary.pdf]

Combined plasma DHA-containing phosphatidylcholine PCaa C38:6 and tetradecanoyl-carnitine as an early biomarker for assessing the mortality risk among sarcopenic patients

Hung-Yao Ho [1,2,3,4], Yun-Ho Chen [3], Chi-Jen Lo [1,2], Hsiang-Yu Tang [1] , Su-Wei Chang [5,6], Chun-Ming Fan [1], Yu-Hsuan Ho [7], Gigin Lin [2,8,9], Chih-Yung Chiu [2,10,11], Chih-Ming Lin [12,13,14,*] and Mei-Ling Cheng [1,2,7,*]

[1] Metabolomics Core Laboratory, Healthy Aging Research Center, Chang Gung University, Taoyuan, Taiwan; hoh01@mail.cgu.edu,.tw (H.-Y. H.); chijenlo@mail.cgu.edu.tw (C.-J. L.); tangshyu@gmail.com (H.-Y.T.); jimmyf@gap.cgu.edu.tw (C.-M. F.); chengm@gap.cgu.edu.tw (M.-L.C.)
[2] Clinical Metabolomics Core Laboratory, Chang Gung Memorial Hospital at Linkou, Taoyuan, Taiwan; giginlin@cgmh.org.tw (G.L.); pedchestic@gmail.com (C.-Y.C.)
[3] Graduate Institute of Biomedical Sciences, College of Medicine, Chang Gung University, Taoyuan, Taiwan; david820524@gmail.com (Y.-H.C.)
[4] Department of Medical Biotechnology and Laboratory Science, College of Medicine, Chang Gung University, Taoyuan, Taiwan
[5] Clinical Informatics and Medical Statistics Research Center, Chang Gung University, Taoyuan, Taiwan; shwchang@mail.cgu.edu.tw (S.-W.C.)
[6] Department of Laboratory Medicine, Chang Gung Memorial Hospital at Linkou, Taoyuan, Taiwan
[7] Department of Biomedical Sciences, College of Medicine, Chang Gung University, Taoyuan, Taiwan; hokylie4@gmail.com (Y.-H.H.)
[8] Department of Medical Imaging and Intervention, Chang Gung Memorial Hospital at Linkou and Chang Gung University, Taoyuan, Taiwan
[9] Department of Medical Imaging and Radiological Sciences, Chang Gung University, Taoyuan, Taiwan
[10] Department of Pediatrics, Chang Gung Memorial Hospital at Linkou, and Chang Gung University, Taoyuan, Taiwan
[11] Department of Pediatrics, Chang Gung Memorial Hospital at Keelung, and Chang Gung University, Taoyuan, Taiwan
[12] Division of Internal Medicine, Chang Gung Memorial Hospital at Taipei, Taipei, Taiwan; lin789@adm.cgmh.org.tw (C.-M.L.)
[13] Department of Health Management, Chang Gung Health and Culture Village, Taoyuan, Taiwan
[14] College of Medicine, Chang Gung University, Taoyuan, Taiwan
* Correspondence: chengm@mail.cgu.edu.tw; Tel.: +886-3-2118800 ext. 3811 (M.-L.C.); lin789@adm.cgmh.org.tw (C.-M.L.)

Supplemental Material includes three Supplemental Tables and four Supplemental Figure

Supplemental Tables

Table S1. The 5.5-year mortality of all participants.

Table S2. Differentially abundant metabolites in plasma of the Alive and Dead cohorts of non-sarcopenic subjects and sarcopenic patients.

Table S3. The mean plasma concentrations of PCaa C38:6 and C14-carnitine and the mortality of sarcopenic patients grouped on the basis of tertile concentrations and hypertension status.

Supplemental Figures

Figure S1. The study flow diagram. Two hundred and thirty-four participants were enrolled in the present study, and plasma samples were collected. Of these participants, 11 cancer patients were excluded. Forty-four normal subjects, 81 non-sarcopenic LPF subjects, and 98 sarcopenic patients were categorized according to the AWGS 2019 criteria. The normal and non-sarcopenic LPF subjects were grouped as non-sarcopenic subjects. Plasma samples were subjected to metabolomic analyses. All subjects were followed up for 5.5 years. One hundred and six non-sarcopenic subjects were (group 1) alive, and 10 (group 2) deceased. Fifty-four sarcopenic patients (group 3) were alive, and 35 (group 4) died.

Figure S2. Global analysis of plasma metabolites in sarcopenic and non-sarcopenic patients. Plasma collected from the non-sarcopenia alive (group 1), non-sarcopenia dead (group 2), sarcopenia alive (group 3) and sarcopenia dead (group 4) patient cohorts (as defined in Figure S1) were subjected to global metabolomic analysis. A total of 3276 unique features were identified. The datasets were analyzed using PLS-DA. Fifty-three metabolites had VIP scores greater than 3. The datasets were independently analyzed using ANOVA with Tukey's HSD test. Thirty-nine differentially abundant metabolites were found ($P < 0.05$). The Venn diagram shows that only 4 metabolites fit both criteria. These metabolites were identified to be the [M+H] and [M+Na] adducts of PCaa C38:6 and PCaa C40:6.

Figure S3. The proportion of different molecular species with formulae PCaa C38:6 and PCaa C40:6. The plasma samples were analyzed using TOF-MS/MS for identification and quantification of the molecular species with respective chemical formulae. The proportion was calculated accordingly. Data are mean ± SD, N = 86.

Figure S4. The Kaplan-Meier survival curves of the non-sarcopenic (*N*) or sarcopenic (*S*) patients without (*Non-HTN*) or with hypertension (*HTN*). (A,B) All participants were categorized into N and S cohorts (A), or non-HTN and HTN cohorts (B). (C,D) All participants were categorized into non-HTN (C) or HTN (D) cohorts, each of which was further subdivided into N and S cohorts.

Table S1. The 5.5-year mortality of all participants.

A. The mortality of the non-sarcopenia, low physical function, and sarcopenia cohorts.

	Non-sarcopenia		Sarcopenia
	Normal	LPF	
	N (%)	N (%)	N (%)
Alive	38 (86.4%)	68 (84.0%)	54 (55.1%)
Bedridden	2 (4.5%)	7 (8.6%)	9 (9.2%)
Dead	4 (9.1%)	6 (7.4%)	35 (35.7%)

B. The mortality of the non-sarcopenia and sarcopenia cohorts.

	Non-sarcopenia	Sarcopenia
	N (%)	N (%)
Alive	106 (84.8%)	54 (55.1%)
Bedridden	9 (7.2%)	9 (9.2%)
Dead	10 (8.0%)	35 (35.7%)

The normal and non-sarcopenic LPF subjects were included in the non-sarcopenia cohort.

Table S2. Differentially abundant metabolites in plasma of the Alive and Dead cohorts of non-sarcopenic subjects and sarcopenic patients.

	Non-sarcopenia			Sarcopenia		
Metabolite	Alive, N = 106	Dead, N = 10	P-value[1]	Alive, N = 54	Dead, N = 35	P-value[1]
C4-OH (C3-DC)	0.062 ± 0.027	0.061 ± 0.016	0.92290	0.058 ± 0.018	0.073 ± 0.023	0.0005
C4	0.245 ± 0.129	0.234 ± 0.045	0.58703	0.237 ± 0.067	0.291 ± 0.112	0.0138
C6 (C4:1-DC)	0.108 ± 0.037	0.110 ± 0.022	0.88342	0.114 ± 0.035	0.143 ± 0.057	0.0098
C5:1-DC	0.014 ± 0.005	0.013 ± 0.001	0.15248	0.014 ± 0.006	0.017 ± 0.008	0.0407
C5-DC (C6-OH)	0.033 ± 0.013	0.032 ± 0.005	0.42598	0.032 ± 0.009	0.043 ± 0.018	0.0036
C9	0.029 ± 0.013	0.026 ± 0.005	0.08253	0.029 ± 0.009	0.035 ± 0.015	0.0240
C10	0.331 ± 0.136	0.358 ± 0.110	0.54943	0.355 ± 0.127	0.425 ± 0.176	0.0457
C12	0.115 ± 0.035	0.115 ± 0.024	0.96632	0.117 ± 0.027	0.138 ± 0.037	0.0045
C14	0.035 ± 0.006	0.036 ± 0.003	0.55319	0.035 ± 0.006	0.040 ± 0.006	0.0002
C14:1-OH	0.016 ± 0.004	0.017 ± 0.004	0.85965	0.017 ± 0.004	0.019 ± 0.004	0.0495
C16-OH	0.007 ± 0.002	0.008 ± 0.001	0.35751	0.007 ± 0.001	0.008 ± 0.001	0.0129
C16:1-OH	0.012 ± 0.002	0.012 ± 0.002	0.51024	0.012 ± 0.002	0.013 ± 0.002	0.0389
Cit	39.759 ± 17.286	38.150 ± 10.685	0.77354	41.459 ± 11.550	48.817 ± 15.037	0.0108
Ser	124.295 ± 20.761	131.260 ± 25.588	0.32235	125.533 ± 18.779	112.406 ± 24.929	0.0058
Trp	57.322 ± 11.552	56.840 ± 11.690	0.90000	59.626 ± 10.479	54.169 ± 12.278	0.0275
Creatinine	97.242 ± 81.763	93.480 ± 29.455	0.76109	96.309 ± 32.105	130.029 ± 63.307	0.0055
Kynurenine	2.382 ± 0.664	2.558 ± 0.596	0.41974	2.508 ± 0.700	2.863 ± 0.926	0.0426
SDMA	0.760 ± 0.417	0.799 ± 0.180	0.58665	0.789 ± 0.257	1.120 ± 0.538	0.0014
lysoPC a C18:0	41.400 ± 8.852	34.660 ± 10.318	0.02511	41.648 ± 9.090	35.900 ± 10.939	0.0086
lysoPC a C24:0	0.247 ± 0.050	0.222 ± 0.037	0.11862	0.248 ± 0.050	0.225 ± 0.051	0.0375
lysoPC a C26:0	0.177 ± 0.043	0.170 ± 0.034	0.60279	0.168 ± 0.031	0.153 ± 0.026	0.0225
PC aa C36:1	32.679 ± 7.843	34.680 ± 12.815	0.63836	31.363 ± 7.949	27.626 ± 9.479	0.0478
PC aa C36:2	198.002 ± 29.423	189.500 ± 34.342	0.39090	195.074 ± 28.864	176.314 ± 33.369	0.0060
PC aa C38:0	3.540 ± 0.946	3.834 ± 1.437	0.54020	3.685 ± 1.187	3.054 ± 1.128	0.0145
PC aa C38:4	85.992 ± 24.350	77.660 ± 23.016	0.30109	84.470 ± 22.484	73.114 ± 20.429	0.0180
PC aa C38:5	40.739 ± 10.736	39.050 ± 17.050	0.76497	40.076 ± 11.407	33.763 ± 10.186	0.0094
PC aa C38:6	88.734 ± 23.968	92.170 ± 32.817	0.67591	85.576 ± 22.647	66.229 ± 20.437	0.0001
PC aa C40:5	8.330 ± 2.259	7.642 ± 2.570	0.36478	7.757 ± 1.902	6.541 ± 1.826	0.0036
PC aa C40:6	32.839 ± 9.150	33.620 ± 14.030	0.86645	30.774 ± 7.525	23.278 ± 7.337	0.0000
PC ae C30:2	0.067 ± 0.015	0.069 ± 0.020	0.67346	0.064 ± 0.014	0.057 ± 0.013	0.0195
PC ae C36:2	10.045 ± 2.263	10.753 ± 2.907	0.35802	10.234 ± 2.190	9.083 ± 1.862	0.0120
PC ae C38:0	1.754 ± 0.678	1.732 ± 0.728	0.92300	1.688 ± 0.540	1.298 ± 0.530	0.0012
PC ae C38:2	0.740 ± 0.301	0.814 ± 0.485	0.64638	0.811 ± 0.364	0.667 ± 0.237	0.0266
PC ae C38:6	6.680 ± 1.822	6.648 ± 2.264	0.95863	6.764 ± 2.319	5.778 ± 2.123	0.0461
PC ae C40:1	1.030 ± 0.280	0.986 ± 0.294	0.63165	1.073 ± 0.283	0.833 ± 0.246	0.0001

PC ae C40:6	4.171 ± 0.948	4.393 ± 1.087	0.48517	4.271 ± 1.092	3.681 ± 1.074	0.0142
PC ae C42:0	0.786 ± 0.164	0.819 ± 0.129	0.54065	0.813 ± 0.152	0.735 ± 0.164	0.0241
PC ae C42:1	0.356 ± 0.080	0.369 ± 0.063	0.62498	0.372 ± 0.087	0.322 ± 0.067	0.0047
PC ae C42:2	0.489 ± 0.117	0.480 ± 0.122	0.83062	0.502 ± 0.127	0.437 ± 0.120	0.0168
PC ae C42:3	0.738 ± 0.175	0.815 ± 0.192	0.19066	0.781 ± 0.194	0.697 ± 0.184	0.0457
SM (OH) C22:1	49.325 ± 10.752	51.520 ± 13.016	0.54557	49.841 ± 9.998	41.783 ± 8.431	0.0002
SM (OH) C22:2	52.066 ± 12.564	55.070 ± 11.672	0.46890	52.750 ± 9.190	44.994 ± 10.906	0.0005
SM (OH) C24:1	2.364 ± 0.541	2.737 ± 0.580	0.04051	2.365 ± 0.495	2.131 ± 0.414	0.0227
SM C16:1	31.457 ± 6.015	32.740 ± 7.399	0.52843	32.228 ± 6.778	28.869 ± 7.027	0.0269
SM C18:1	22.880 ± 5.132	22.550 ± 5.268	0.84645	21.754 ± 4.441	19.175 ± 5.373	0.0158
SM C24:0	51.067 ± 11.017	55.360 ± 13.876	0.25190	51.783 ± 10.796	45.971 ± 10.365	0.0136

Values are mean ± SD. [1]*Alive* vs. *Dead* cohorts of the same subject cohort. C4-OH (C3-DC): hydroxybutyrylcarnitine (malonylcarnitine); C4: butyrylcarnitine; C6 (C4:1-DC): hexanoylcarnitine (fumarylcarnitine); C5:1-DC: glutaconylcarnitine; C5-DC (C6-OH): glutarylcarnitine (hydroxyhexanoylcarnitine); C9: nonaylcarnitine; C10: decanoylcarnitine; C12: dodecanoylcarnitine; C14: tetradecanoylcarnitine; C14:1-OH: hydroxytetradecenoylcarnitine; C16-OH: hydroxyhexadecanoylcarnitine; C16:1-OH: hydroxyhexadecenoylcarnitine; Cit: citrulline; Ser: serine; Trp: tryptophan; SDMA: symmetric dimethylarginine; LysoPCa: lysophosphatidylcholine (acyl; a fatty acid is linked via an ester bond); PCaa: phosphatidylcholine (diacyl; both fatty acids are linked via ester bonds); PCae: phosphatidylcholine (a fatty acid is linked via an ester bond, while another is linked via an ether bond); SM: sphingomyelin; SM (OH): hydroxysphingomyelin.

Table S3. The mean plasma concentrations of PCaa C38:6 and C14-carnitine and the mortality of sarcopenic patients grouped on the basis of tertile concentrations and hypertension status.

A. Mean plasma PCaa C38:6 concentrations of the low (L), mid (M), high (H) tertile groups of sarcopenic patients.

Tertile group*	Sarcopenia, N = 89	Non-HTN, N = 43	HTN, N = 46	Non-HTN vs HTN
PCaa C38:6	Mean ± SD	Mean ± SD	Mean ± SD	P-value
L	53.92 ± 8.13	53.44 ± 9.35	54.44 ± 6.89	0.748
M	75.48 ± 4.88	75.52 ± 4.68	75.45 ± 5.17	0.973
H	103.70 ± 18.55	107.29 ±22.05	100.11 ± 14.10	0.297

B. The mortality of all, non-HTN and HTN individuals within the low (L), mid (M), high (H) PCaa C38:6 tertile groups of sarcopenic patients.

Tertile group*	Sarcopenia, N = 89	Non-HTN, N = 43	HTN, N = 46	Non-HTN vs HTN
PCaa C38:6	Dead, N (%)	Dead, N (%)	Dead, N (%)	P-value
L (N = 29)	20 (68.97%)	11 (37.93%)	9 (31.03%)	0.614
M (N = 30)	12 (40%)	1 (3.33%)	11 (36.66%)	0.001
H (N = 30)	3 (10%)	0 (0%)	3 (25%)	0.072

C. Mean plasma C14-carnitine concentrations of the low (L), mid (M), high (H) tertile groups of sarcopenic patients.

Tertile group*	Sarcopenia, N = 89	Non-HTN, N = 43	HTN, N = 46	Non-HTN vs HTN
C14, nM	Mean ± SD	Mean ± SD	Mean ± SD	P-value
L	30.37 ± 2.36	29.93 ± 1.94	30.75 ± 2.67	0.35
M	36.35 ± 1.52	35.77 ± 1.24	36.92 ± 1.61	0.051
H	43.82 ± 4.90	41.75 ± 4.99	45.76 ± 4.05	0.016

D. The mortality of all, non-HTN and HTN individuals within the low (L), mid (M), high (H) C14-carnitine tertile groups of sarcopenic patients.

Tertile group*	Sarcopenia, N = 89	Non-HTN, N = 43	HTN, N = 46	Non-HTN vs HTN
C14, nM	Dead, N (%)	Dead, N (%)	Dead, N (%)	P-value
L (N = 30)	5 (16.67%)	0 (0%)	5 (16.67%)	0.045
M (N = 26)	12 (46.15%)	4 (15.38%)	8 (30.77%)	0.238
H (N = 33)	18 (54.54%)	8 (24.24%)	10 (30.30%)	0.732

*The tertile groups were defined on the basis of concentrations of PCaa C38:6 or C14-carnitine in plasma of sarcopenic patients.

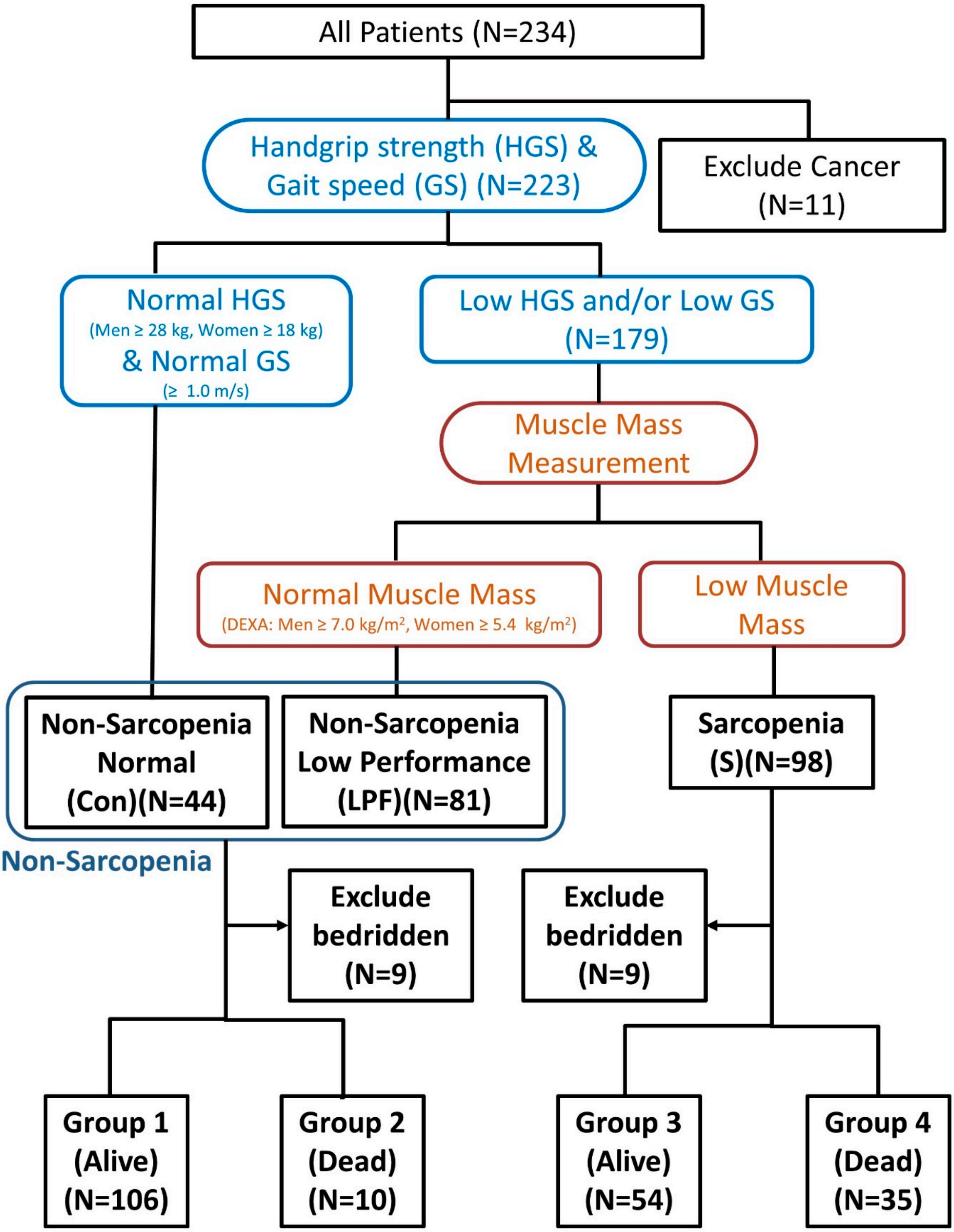

Figure S1. The study flow diagram. Two hundred and thirty-four participants were enrolled in the present study, and plasma samples were collected. Of these participants, 11 cancer patients were excluded. Forty-four normal subjects, 81 non-sarcopenic LPF subjects, and 98 sarcopenic patients were categorized according to the AWGS 2019 criteria. The normal and non-sarcopenic LPF subjects were grouped as non-sarcopenic subjects. Plasma samples were subjected to metabolomic analyses. All subjects were followed up for 5.5 years. One hundred and six non-sarcopenic subjects were (group 1) alive, and 10 (group 2) deceased. Fifty-four sarcopenic patients (group 3) were alive, and 35 (group 4) died.

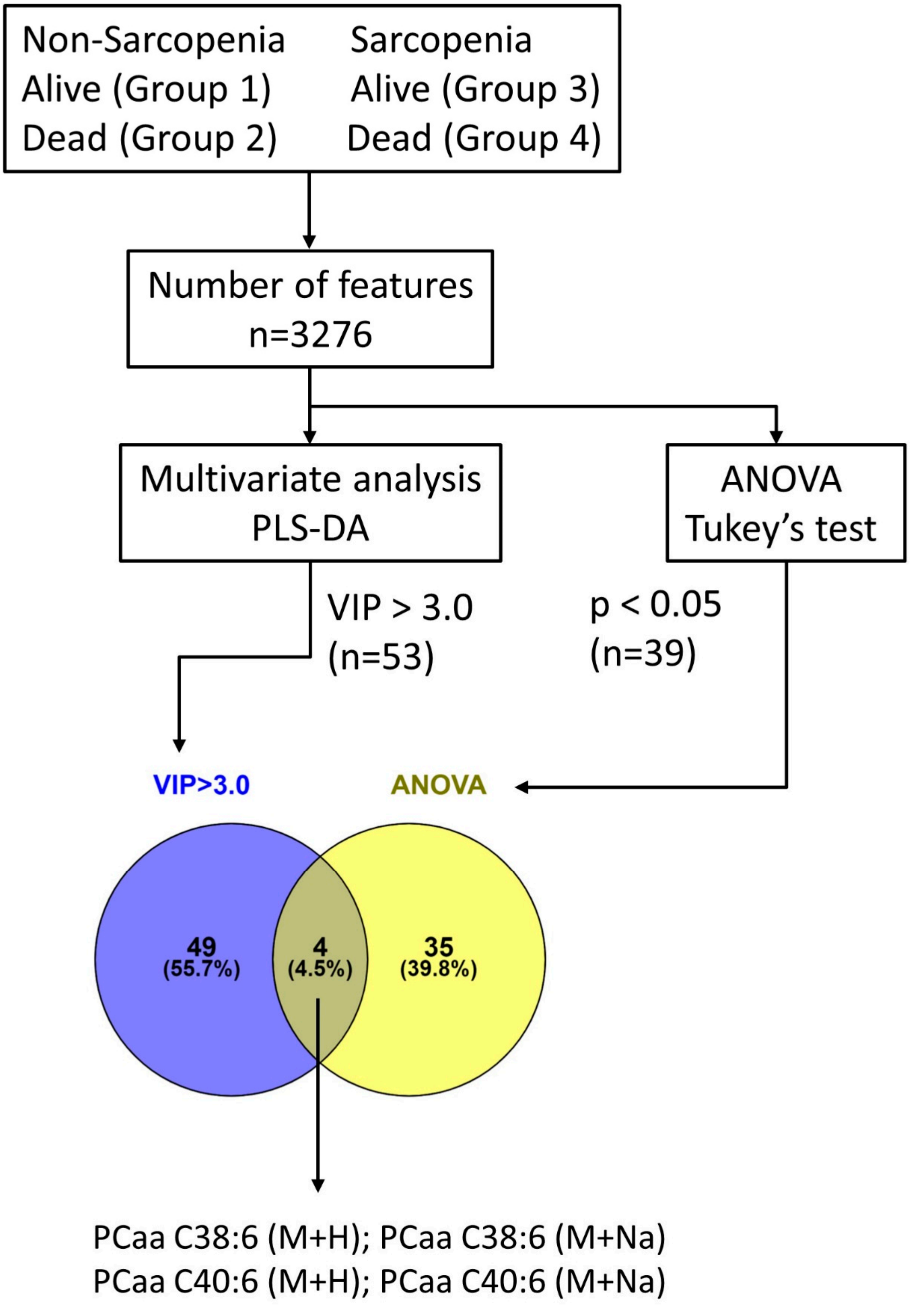

Figure S2. Global analysis of plasma metabolites in sarcopenic and non-sarcopenic patients. Plasma collected from the non-sarcopenia alive (group 1), non-sarcopenia dead (group 2), sarcopenia alive (group 3) and sarcopenia dead (group 4) patient cohorts (as defined in Figure S1) were subjected to global metabolomic analysis. A total of 3276 unique features were identified. The datasets were analyzed using PLS-DA. Fifty-three metabolites had VIP scores greater than 3. The datasets were independently analyzed using ANOVA with Tukey's HSD test. Thirty-nine differentially abundant metabolites were found ($P < 0.05$). The Venn diagram shows that only 4 metabolites fit both criteria. These metabolites were identified to be the [M+H] and [M+Na] adducts of PCaa C38:6 and PCaa C40:6.

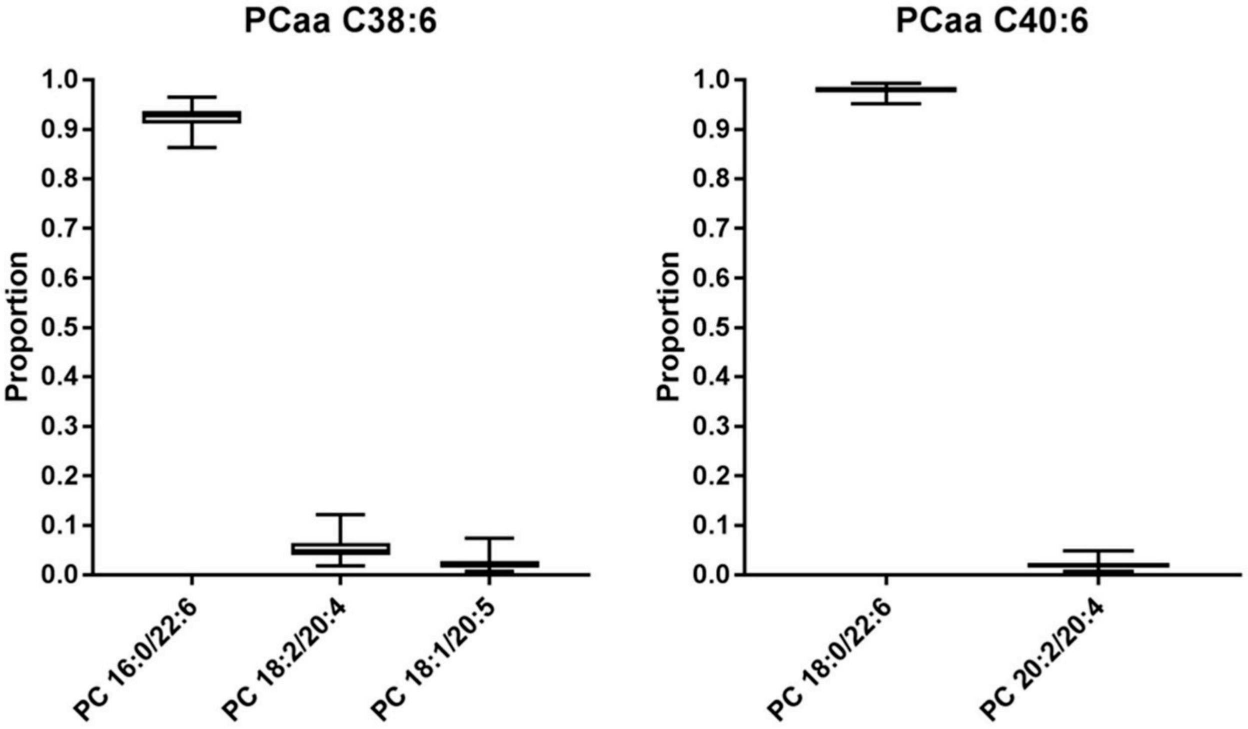

Figure S3. The proportion of different molecular species with formulae PCaa C38:6 and PCaa C40:6. The plasma samples were analyzed using TOF-MS/MS for identification and quantification of the molecular species with respective chemical formulae. The proportion was calculated accordingly. Data are mean ± SD, N = 86.

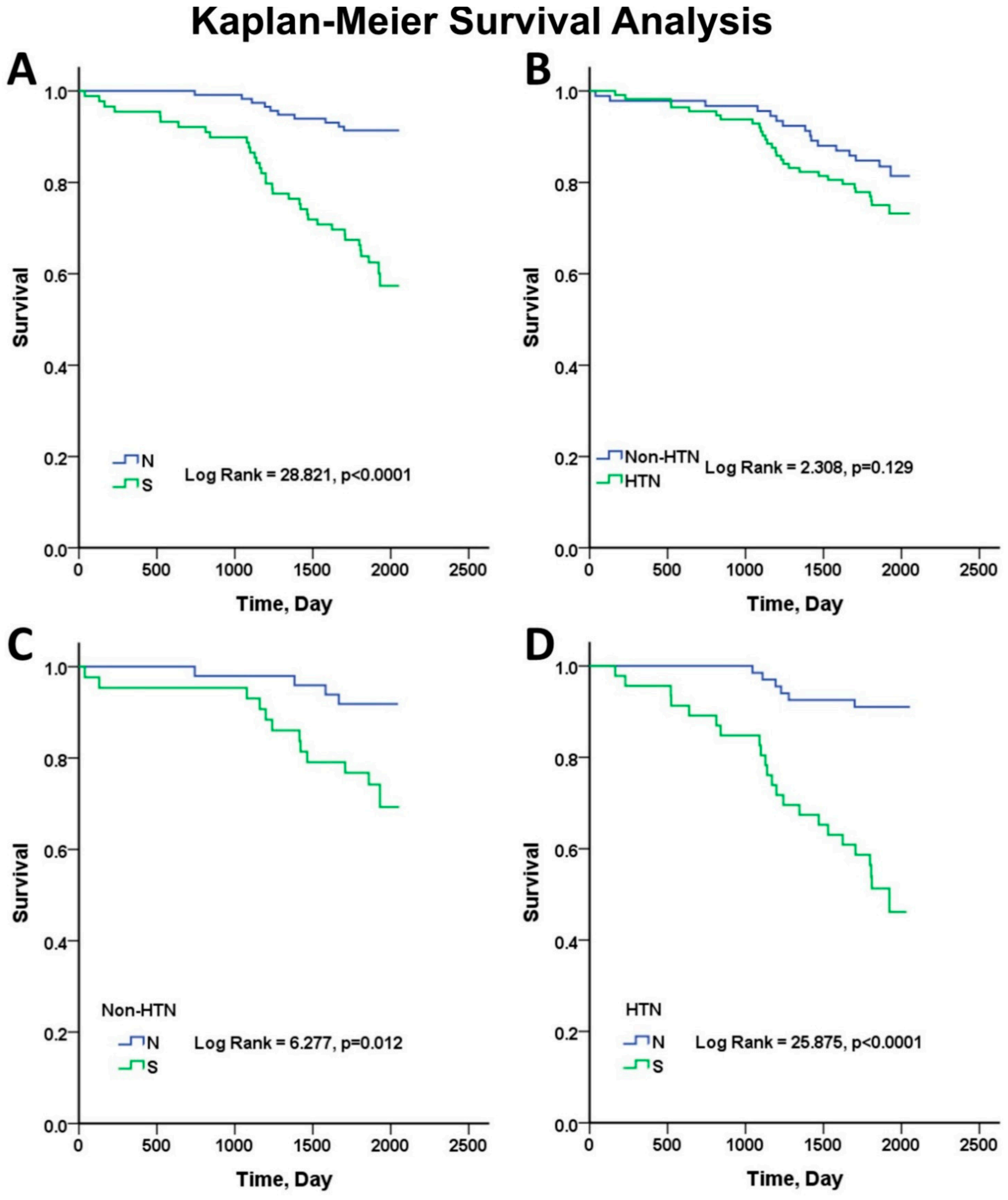

Figure S4. The Kaplan-Meier survival curves of the non-sarcopenic (*N*) or sarcopenic (*S*) patients without (*Non-HTN*) or with hypertension (*HTN*). (A,B) All participants were categorized into N and S cohorts (A), or non-HTN and HTN cohorts (B). (C,D) All participants were categorized into non-HTN (C) or HTN (D) cohorts, each of which was further subdivided into N and S cohorts.
